# Determinants of full immunization coverage among children 12–23 months of age from deviant mothers/caregivers in Ethiopia: A multilevel analysis using 2016 demographic and health survey

**DOI:** 10.3389/fpubh.2023.1085279

**Published:** 2023-02-28

**Authors:** Samrawit Mihret Fetene, Wubshet Debebe Negash, Ever Siyoum Shewarega, Desale Bihonegn Asmamaw, Daniel Gashaneh Belay, Rediet Eristu Teklu, Fantu Mamo Aragaw, Tewodros Getaneh Alemu, Habitu Birhan Eshetu, Elsa Awoke Fentie

**Affiliations:** ^1^Department of Health Systems and Policy, Institute of Public Health, College of Medicine and Health Sciences, University of Gondar, Gondar, Ethiopia; ^2^Department of Reproductive Health, School of Public Health, College of Medicine and Health Sciences, Dilla University, Dilla, Ethiopia; ^3^Department of Reproductive Health, Institute of Public Health, College of Medicine and Health Sciences, University of Gondar, Gondar, Ethiopia; ^4^Department of Human Anatomy, College of Medicine and Health Sciences, University of Gondar, Gondar, Ethiopia; ^5^Department of Epidemiology and Biostatistics, Institute of Public Health, College of Medicine and Health Sciences, University of Gondar, Gondar, Ethiopia; ^6^Department of Pediatrics and Child Health Nursing, School of Nursing, College of Medicine and Health Sciences, University of Gondar, Gondar, Ethiopia; ^7^Department of Health Promotion and Health Behavior, Institute of Public Health, College of Medicine and Health Sciences, University of Gondar, Gondar, Ethiopia

**Keywords:** full immunization, positive deviance, multilevel-mixed effect, Ethiopia, EDHS 2016

## Abstract

**Background:**

Despite remarkable improvements in child health services utilization, childhood immunization has been poorly implemented in Ethiopia. However, evidence on the coverage of immunization among children from mothers/caregivers with no education (non-educated mothers were the most identified risk for underutilization of services) are scarce. Therefore, this study aimed to assess the determinants of full immunization coverage among children 12–23 months of age from deviant mothers/caregivers in Ethiopia.

**Methods:**

We analyzed data from the 2016 Ethiopia Demographic and Health Survey (EDHS) on a sample of 1,170 children 12–23 months of age identified from deviant mothers/caregivers (mothers/caregivers with no education) through a two-stage stratified sampling. A multilevel mixed-effect binary logistic regression analysis was used to identify the individual and community level determinants of full immunization coverage among children 12–23 months of age with their deviant mothers/caregivers. In the final model, a *p*-value of < 0.05 and adjusted odds ratio (AOR) with 95% confidence interval (CI) were used to select statistically significant determinants of full immunization coverage.

**Results:**

The overall full immunization coverage among children 12–23 months of age identified from deviant mothers/caregivers was 27.4% (95%CI: 25.0, 31.0) in Ethiopia. Deviant mothers/caregivers who are employed (AOR = 1.69, 95%CI: 1.68, 2.45), being in the rich household wealth status (AOR = 2.54, 95%CI: 1.53, 4.22), residing in city (AOR = 5.69, 95%CI: 2.39, 13.61), having one to three (AOR: 3.28, 95% CI: 2.12–5.07) and four and more ANC follow-up during the recent pregnancy (AOR: 3.91, 95% CI: 2.45, 6.24) were the determinants that increased full immunization coverage among children 12–23 months of age.

**Conclusions:**

Full immunization coverage among children 12–23 months of age from non-educated mothers/caregivers was low and far behind the national target of coverage. Therefore, a system-wide intervention should be used to enhance employability, wealth status, and key maternal health services like ANC follow-up among non-educated mothers/caregivers to increase their children's full immunization coverage.

## Background

Immunization has been proven to be one of the most cost-effective health interventions in the world, having successfully averted or eradicated severe childhood diseases (1). Childhood vaccination continues to rise substantially across the globe (2). The global under-five mortality rate has decreased by 59% from 93 deaths per 1,000 live births in 1990 to 38 in 2021 (3). Vaccinations have been found to protect children in poor and middle-income countries against preventable diseases- diphtheria, measles, mumps, pertussis, pneumonia, polio, rotavirus diarrhea, rubella, and tetanus (4–6).

Children are considered fully vaccinated by the World Health Organization when they have had one dose of Bacillus Calmette Guerin (BCG), three doses of diphtheria-tetanus-pertussis (DTP3), three doses of the polio vaccine, three doses of Pneumococcal conjugate vaccine (PCV3), 3–4 doses of Hepatitis B, three doses of Haemophilus influenza type b (Hib), 2–3 doses of rotavirus, one doses of rubella and one dose of measles vaccine by the age of 5 years (7). According to WHO data for 2021, 84% of children received BCG, 86% received DTP3, 51% received PCV3, and 81% received measles vaccination globally (8). Vaccination coverage has improved significantly in several African countries in the last few decades (9).

Ethiopia launched expanded immunization program in 1980 with the aim of reducing mortality, morbidity, and disability of children from vaccine preventable diseases (10). The program has been freely provided by the public health sector in collaboration with other non-governmental organizations and donors in all regions and districts though district-based strategies and long-term outreach service approaches to achieve the national targets (11). Despite the free provision of immunization services in Ethiopia, full immunization coverage has not been achieved as expected (12). The total immunization coverage was 24.3% in 2011 (13) and 38.3% in 2016 (14). As a result, in Ethiopia, many children have not received the benefit of full immunization (15).

Previous empirical studies focusing on full immunization coverage among children 12–23 months of age and have revealed determinants of full immunization coverage. The identified individual and community level factors are socio-demographic and economic variables like mothers' educational level, residence, household wealth index, maternal marital and occupational status, religion, region, age, and distance to the nearest health facility (1, 14, 16–23). Previous obstetric characteristics like having an antenatal care (ANC) visit, place of delivery, parity, number of children and childbirth order were also identified (14, 16–18, 20, 24, 25).

Many studies have shown that education is one of the factors significantly associated with full immunization coverage among children (17–19, 26). These studies suggest that improving mothers' education will contribute to improving immunization coverage. However, according to EDHS 2016 report, nearly 50% of women have never attended school in Ethiopia (27) moreover, it is not easy to go back and attend the education for mothers at this stage. Despite accessing the same limited resources, specific individuals or groups (underserved populations) in every community can find better solutions and practices to overcome limited access than their peers—which are a positive deviant (28, 29).

A study conducted in Ghana using the positive deviance (PD) approach showed that even the poorest regions (deviant regions) with disparate characteristics and social situations could achieve excellent child immunization coverage (30). The study suggests future exploration using newer DHS data from countries with district-level data, thereby having much larger sample sizes. But evidence on the determinants of full immunization coverage among children 12–23 months of age from deviant mothers/caregivers are still scarce, as most existing literature focuses on both educated and uneducated (14, 17, 18, 23, 24, 31).

Consequently, in this study, we employed the PD approaches to identify mothers/caregivers with positive health behaviors (determinants of full immunization coverage) despite an adverse profile (being uneducated) using recent DHS data. As a result, knowing the positive determinants of full immunization coverage among children 12–23 months of age from mothers/caregivers with a high risk for immunization services underutilization (being uneducated) could help health policy implementers and enable the EPI program to optimize child health initiative performance, and ultimately improve children's health through vaccine services utilization improvement in resource-limited settings with high illiteracy in Ethiopia. The finding is also essential for designing better strategies to improve full immunization coverage and meet child health related targets of sustainable development goal 3.2. Therefore, this study aimed to assess determinants of full immunization coverage among children 12–23 months of age from deviant mothers/caregivers in Ethiopia.

## Methods

### Data sources and context

This analysis was conducted using a cross-sectional data from the EDHS 2016. The EDHS is a nationally representative household survey implemented by the Central Statistical Agency (CSA) of Ethiopia every 5 years (12). Ethiopia was home to more than 120 million people in 2022, of which 16% were children under 5 years (32). Administratively, the country is divided into nine regions [Tigray, Afar, Amhara, Oromia, Benishangul, Gambela, South Nation Nationalities and Peoples' Region (SNNPR), Harari, and Somali] and two City Administrations (Addis Ababa and Dire-Dawa). These nine regions can be divided into developed regions (Tigray, Amhara, Oromia, SNNPR, and Harari) and emerging regions (Afar, Somalia, Benishangul, and Gambela).

A developed region and city administrations have a relatively dense population, better infrastructure, education services and better accessibility to health, including immunization services (33). In contrast, in emerging regions, where scattered pastoralists are the majority. It is common for emerging regions to suffer from inadequate infrastructure, inaccessible health services, droughts, poverty, and a lack of clear and detailed regulations (34).

### Sample size and sampling procedures

**Figure 1:**
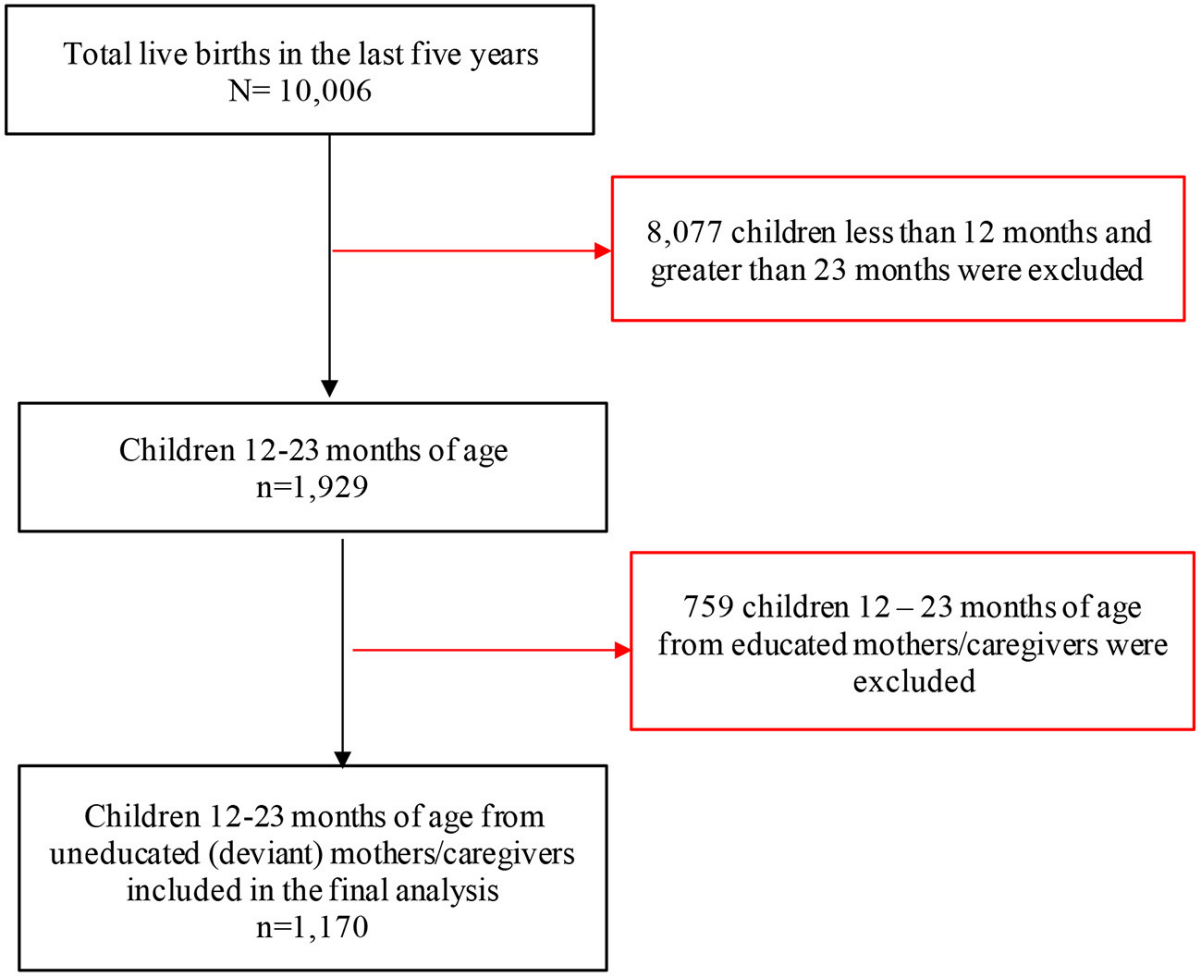
Study sample of children 12–23 months of age with their deviant mothers/caregivers in the 2016 Ethiopia DHS (*n* = 1,170).

The Ethiopian CSA performed a population and housing census in 2007, which was utilized as a sample frame for the 2016 EDHS and provided a complete list of 84,915 enumeration areas (27). To select study participants, the EDHS used a two-stage stratified sampling approach. Each stratum had a sample of EAs, which were chosen at random. Accordingly, all children aged 12–23 months who are regular members of the selected households were eligible for the survey. Finally, a total of 1,170 children 12–23 months of age from deviant mothers/caregivers were identified (Figure 1).

### Identification of positive deviant mothers/caregivers

To identify the positive deviance of mothers/caregivers for full immunization coverage among children aged 12–23 months and determinants of being positive deviant, Anderson's behavioral model of health service (35) and other related studies were used (1, 17, 20, 23, 36).

Accordingly, education is the primary determinant of health services utilization. We selected mothers/caregivers with no formal education as a sub-group with a very low likelihood of fully immunizing their children, as mother/caregiver education was the strongest predictor of full immunization coverage after adjusting for the other risk factors associated with full immunization coverage among children in this population. Positive deviant mothers/caregivers were those who reported no formal education but their children fully immunized. Finally, in the analysis, we compared the characteristics of the PD mothers/caregivers to those of their counterparts. Due to significant variations by clusters in the overall full immunization coverage among children aged 12–23 months of age from deviant mothers/caregivers, analysis was stratified by individual and community level.

### Measurements of variables

The outcome variable for this study was full vaccination coverage among children 12–23 months of age from deviant mothers/caregiver's which is defined as a child who has had one dose of BCG, three doses of pentavalent, three doses of polio, two doses of Rota, three doses of PCV, and one dose of measles (30). If the child had obtained all of the recommended doses of all vaccines, the immunization status was recoded as “1” and classified as “fully immunized”, or if the child had missed one or more doses, the immunization status was recoded as “0” and was classified as “not fully immunized” (14).

Our study assessed independent variables by considering the individual and community-level variables (1, 6, 14, 18, 20, 23). Individual-level variables include, the age of deviant mother/caregivers recoded in completed years (15–24, 25–34, 35+), employment status (employed, non-employed), religion (muslim, orthodox, and other), marital status (married, not married), household wealth status (poor, middle, and rich), head of household (male, female), sex of child (male, female), health insurance coverage (yes, no), i.e., in Ethiopia, community-based health insurance the only health insurance that has been implemented in all regions at household level (37), educational status of husband (no education, primary, secondary, and above), number of ANC visit (no visit, 1–3 visits, 4+ visits), place of delivery (home, health facility), parity (1, 2–5, 6+), childbirth order (1, 2–5, 6+) and uptake of postnatal care (PNC) (yes, no). The uptake of PNC services was assessed whether women received PNC services within 2 months after delivery, regardless of their place of birth. PNC services were assessed based on the mothers/caregiver's verbal responses during the survey. Therefore, it was categorized as “yes” if a woman had at least one PNC visit; otherwise “no.”

The wealth index is a composite measure of a household's cumulative living standard. It is calculated using readily available data on a household's ownership of certain assets, such as televisions and bicycles, materials used for housing construction, and types of water access and sanitation facilities. The household wealth index was originally classified into five categories (poorest, poorer, middle, richer, and richest) by the DHS, which was done with principal component analysis (12). However, for analysis in this study, we divided wealth status into three categories: poor, average, and rich.

On the other hand, the community-level variables include, place of residence (rural, urban), region (emerging region, developed region, and city administration), the difficulty of getting health services (big problem or not big problem) and media exposure. Deviant mothers/caregiver's media exposure was assessed from the three variables: watching television, listening radio, and reading a newspaper, and labeled as “yes” if a woman has exposure to either of the three media sources at least once a week or “no” if a woman has exposure to none of them.

### Data processing and analysis

The STATA software version 16 was used to extract, clean, recode, and analyze the data. The descriptive statistics were presented *via* tables, figures, and narrations. The EDHS data were collected using multistage stratified cluster sampling techniques; as a result, the data had a hierarchical (individuals were nested within communities) nature. Besides, selected and interviewed deviant mothers/caregivers in the same cluster are more likely to be similar to each other than deviant mothers/caregivers from another cluster. This implies that there is a need to consider the between cluster variability by using advanced models. Therefore, to identify determinants, and to estimate the effect of independent variables on full immunization coverage among children 12–23 months of age with their deviant mothers/caregivers, we used the multilevel binary logistic regression analysis method. The Interclass Correlation Coefficient (ICC) and Median Odds Ratio (MOR) were checked to assess whether there was significant clustering or not (38). Accordingly, we found 48% of ICC in our study which showed that 48% of the variation in full coverage among children 12–23 months of age from deviant mothers/caregivers can be explained by clustering.

Four models were fitted in this study—null model (no explanatory variables), model I (individual-level factors), model II (community-level factors), model III (both individual and community-level factors). The ICC and deviance (-2^*^ log-likelihood ratio) were used to evaluate model comparison and fitness. Model III was selected as the best-fitted model since it had the lowest deviance. The proportion of variance (PCV) explained by the grouping structure in the population was calculated to analyze the variation between clusters (39).

In the bivariable analysis, variables with a *p*-value < 0.2 were considered for multivariable analysis in each three models. Finally, adjusted odds ratios (AOR) with 95% CI and *p*-value of ≤ 0.05 in the multivariable analysis were used to declare statistically significant determinants of full immunization coverage among children 12–23 months of age from deviant mothers/caregivers in the final model. Multicollinearity was tested using the variance inflation factor (VIF). There was a VIF of <5 for each independent variable with a mean VIF of 1.85, indicating no significant multicollinearity between independent variables.

## Results

### Characteristics of study participants

A total of 1,170 children 12–23 months of age with their deviant mothers/caregivers were included in this analysis (Figure 1). The socio-demographic and economic characteristics of these deviant mothers/caregivers and their children 12–23 months of age are presented in Table 1. The mean age of mothers/caregivers was 30 ± 6.5 years, 64.1% were in poor household wealth status and 57.18% were Muslim religion followers. Moreover, 95.2% of the mothers/caregivers are married and of these married 66.3% of their husbands were uneducated. The mean age of children 12–23 months of age was 16.7 ± 3.4 months and half of children were males.

**Table 1:** Socio-demographic and economic characteristics of deviant mothers/caregivers of children aged 12–23 months in Ethiopia, EDHS 2016 (*n* = 1,170).

**Variables**	**Frequency**	**Percentage**
**Age of mothers/caregivers (in years)**		
15–24	230	19.66
25–34	626	53.50
≥35	314	26.84
**Religion**		
Muslim	669	57.18
Orthodox	313	26.75
Other	188	16.07
**Employment status**		
Not employed	708	60.51
Employed	462	39.49
**Mothers/caregivers marital status**		
Not married	56	4.79
Married	1,114	95.21
**Educational status of husband**		
Uneducated	739	66.34
Primary school	315	28.28
Secondary and above	60	5.39
**Sex of household head**		
Male	931	79.57
Female	239	20.43
**Household wealth index**		
Poor	750	64.10
Middle	174	14.87
Rich	246	21.03
**Sex of child**		
Male	594	50.77
Female	576	49.23
**Age of child (in months)**		
12–18	791	67.61
19–23	379	32.39
**Household covered by health insurance** ^*^		
No	1,138	97.26
Yes	32	2.74

Other = Protestant, catholic.

* Insurance = in Ethiopia, community-based health insurance the only health insurance that has been implemented in all regions at household level.

### Obstetric characteristics of the deviant mothers/caregivers

Table 2 shows the obstetric characteristics of deviant mothers/caregivers of children aged 12–23 months in Ethiopia. Nearly 45% of deviant mothers/caregivers have not received ANC visits for their recent pregnancy, and 74.5% of the mothers/caregivers gave their recent birth at home. The majority (93.7%) of deviant mothers/caregivers were not received PNC.

**Table 2:** Obstetric characteristics of deviant mothers/caregivers of children aged 12–23 months in Ethiopia, EDHS 2016 (*n* = 1,170).

**Variables**	**Frequency**	**Percentage**
**Numbers of ANC follow up**		
No	496	44.68
1–3	335	30.18
4+	279	25.14
**PNC uptake**		
No	1,041	93.78
Yes	69	6.22
**Place of delivery**		
Home	872	74.53
Health facility	298	25.47
**Parity**		
1	126	10.77
2–5	694	59.32
6+	350	29.91
**Child birth order**		
1	124	10.60
2–5	623	53.25
6+	423	36.15

### Community-level variables for full immunization coverage

The description of community-level factors of full immunization in Ethiopia are presented in Table 3. In this analysis, only 21.45% of deviant mothers/caregivers had access to all media types (radio, newsletter, and television) more than once a week, and 90.2% were rural dwellers. Nearly 40% of the deviant mothers/caregivers were living closer to health facility.

**Table 3:** Health services related and community-level characteristics of deviant mothers/caregivers of children aged 12–23 months in Ethiopia, EDHS 2016 (*n* = 1,170).

**Variables**	**Frequency**	**Percentage**
**Residence**		
Rural	1,055	90.17
Urban	115	9.83
**Media exposure**		
Low	919	78.55
High	251	21.45
**Region**		
Emerging	489	41.79
Developed	614	52.48
City administration	67	5.73
**Distance to the health facility**		
Big problem	709	60.60
Not big problem	461	39.40

### Full immunization coverage among children 12–23 months of age from deviant mothers/caregivers

**Figure 2:**
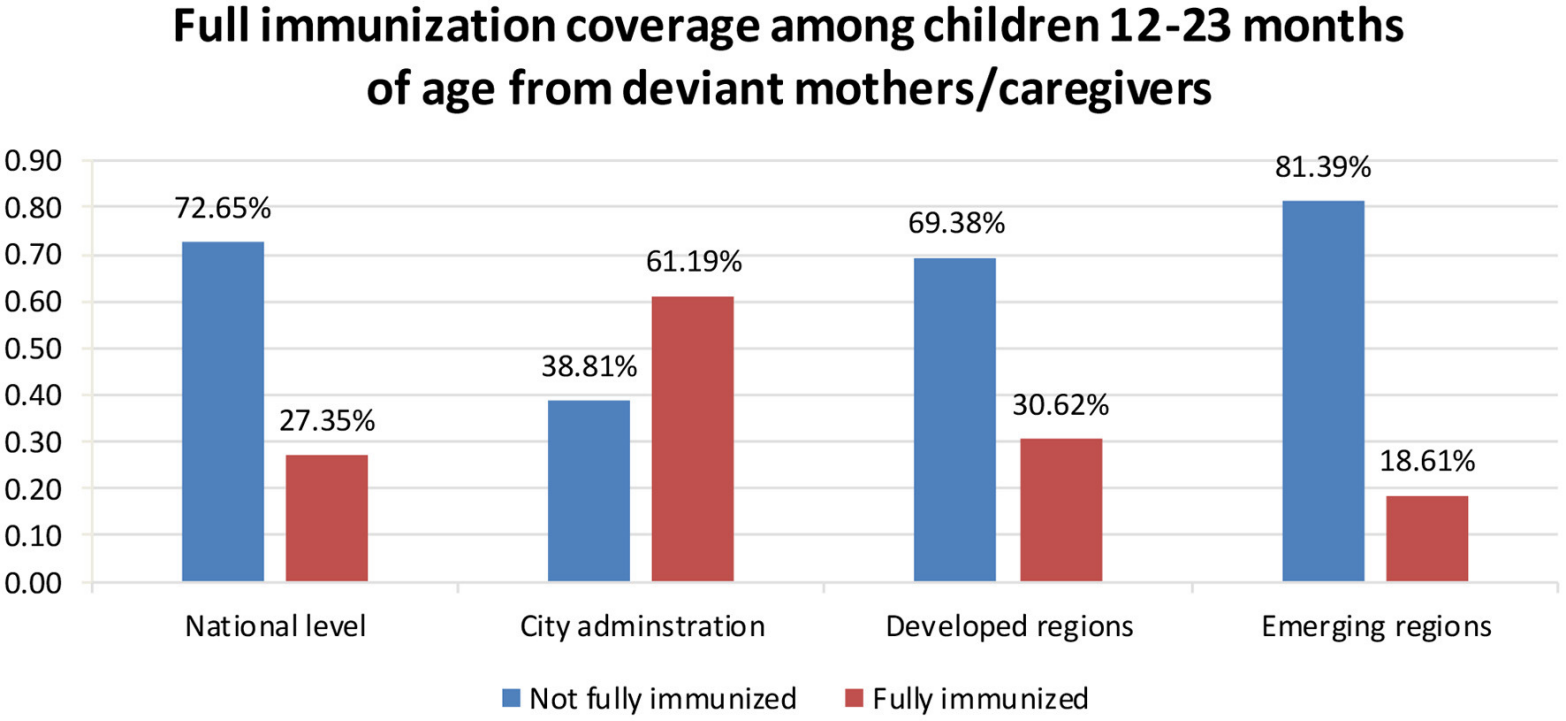
Full immunization coverage among children 12–23 months of age from deviant mothers/caregivers in the 2016 Ethiopia DHS (*n* = 1,170).

The overall full immunization coverage among children 12–23 months of age from deviant mothers/caregivers in Ethiopia was 27.4% (95%CI: 25.0, 31.0). Of those fully immunized, 18.1, 30.6, and 38.8% of children with their deviant mothers/caregivers were residing in emerging regions, developed regions, and city administrations, respectively (Figure 2).

### Measure of variation using random effects and model fitness

There was a significant variation in full immunization coverage among children 12–23 months of age from deviant mothers/caregivers across clustering. The model fitness was checked using the ICC across, Akaike's Information Criterion (AIC) and deviance as presented in Table 4. Then model three (a model with both individual and community level factors) was best (low deviance and AIC) and chosen for the final analysis to identify the determinants of full immunization coverage.

**Table 4:** Random-intercept model of multilevel analysis for full immunization coverage among children 12–23 months of age from deviant mothers/caregivers in Ethiopia, EDHS 2016.

**Measures of variation**	**Null-model**	**Model-one**	**Model-two**	**Model-three**
**Variance**				
ICC	0.48	0.22	0.36	0.20
MOR	3.98 (3.12–5.05)	2.49 (1.69–3.68)	3.61 (2.81–4.64)	2.39 (1.59–3.62)
**Model fitness**				
Deviance (-2^*^LLR)	1,305	1,066	1,245	1,050
AIC	1,309	1,115	1,257	1,104

### Factors affecting full immunization coverage among children 12–23 months of age from deviant mothers/caregivers

After adjusting for individual and community level factors, deviant mothers/caregivers who are employed, rich household wealth status, had ANC follow-up, and residing in city administrations were statistically significant determinants of full immunization coverage as presented in Table 5.

**Table 5:** A mixed effect multilevel logistic regression analysis of individual and community-level factors associated with full immunization coverage among deviant mothers/caregiver's children aged 12–23 months in Ethiopia, EDHS 2016 (*n* = 1,170).

**Variables**	**Full immunization coverage**		**COR (95%CI)**	**Model-I AOR (95%CI)**	**Model-II** ** AOR (95%CI)**	**Model-III AOR (95%CI)**
	**Yes (%)**	**No (%)**				
**Age of mothers/caregivers (in year)**						
15–24	58 (25.22)	172 (74.78)	0.97 (0.59, 1.61)	1.08 (0.54, 2.16)		1.16 (0.58, 2.33)
25–34	174 (27.80)	452 (72.20)	1.14 (0.77, 1.70)	1.07 (0.67, 1.69)		1.11 (0.69, 1.76)
≥35	88 (28.03)	226 (71.97)	1	1		1
**Employment status**						
Not employed	158 (22.32)	550 (77.68)	1	1		1
Employed	162 (35.06)	300 (64.94)	1.98 (1.39, 2.84)	1.57 (1.09, 2.28)		1.69 (1.68, 2.45)^*^
**Religion**						
Muslim	159 (23.77)	510 (76.23)	1	1		1
Orthodox	122 (38.98)	191 (61.02)	2.37 (1.48, 3.79)	1.45 (0.91, 2.31)		1.52 (0.92, 2.52)
Others	39 (20.74)	149 (79.26)	0.86 (0.48, 1.55)	0.86 (0.49, 1.49)		0.95 (0.54, 1.69)
**Educational status of husband**						
Uneducated	177 (23.95)	562 (76.05)	1	1		1
Primary	110 (34.92)	205 (65.08)	1.68 (1.15, 2.44)	1.14 (0.78, 1.69)		1.04 (0.70, 1.54)
Secondary and above	18 (30.0)	42 (70.0)	1.38 (0.65, 291)	0.72 (0.32, 1.61)		0.76 (0.34, 1.72)
**Sex of household head**						
Male	273 (29.32)	658 (70.68)	1	1		1
Female	47 (19.67)	192 (80.33)	0.68 (0.43, 1.08)	0.73 (0.44, 1.21)		0.78 (0.47, 1.31)
**Wealth index**						
Poor	153 (20.40)	597 (79.60)	1	1		1
Middle	57 (32.76)	117 (67.24)	1.71 (1.06, 2.75)	1.26 (0.78, 2.04)		1.21 (0.75, 1.98)
Rich	110 (44.72)	136 (55.28)	4.62 (2.93, 7.29)	2.84 (1.79, 4.50)		2.54 (1.53, 4.22)^*^
**Sex of child**						
Male	160 (26.94)	434 (73.06)	0.88 (0.63, 1.23)	0.97 (0.69, 1.36)		0.99 (0.71, 1.40)
Female	160 (27.78)	416 (72.22)	1	1		1
**Covered by health insurance**						
No	304 (26.71)	834 (73.29)	1	1		1
Yes	16 (50)	16 (50)	2.27 (0.84, 6.10)	1.10 (0.43, 2.84)		1.12 (0.44, 2.88)
**Number of ANC follow up**						
No	63 (12.70)	433 (87.30)	1	1		1
1–3	111 (33.13)	224 (66.87)	3.67 (2.43, 5.54)	3.21 (2.09, 4.94)		3.28 (2.12, 5.07)^*^
≥4	131 (46.95)	148 (53.05)	6.29 (4.13, 9.59)	4.23 (2.65, 6.73)		3.91 (2.45, 6.24)^*^
**PNC**						
No	272 (26.13)	769 (73.87)	1	1		1
Yes	33 (47.83)	36 (52.17)	2.79 (1.43, 5.44)	1.37 (0.72, 2.63)		1.46 (0.76, 2.81)
**Place of delivery**						
Home	192 (22.02)	680 (77.98)	1	1		1
Health facility	128 (42.95)	170 (57.05)	2.63 (1.81, 3.81)	1.26 (0.84, 1.89)		1.17 (0.77, 1.78)
**Parity**						
1	35 (27.78)	91 (72.22)	1	1		1
2-5	193 (27.81)	501 (72.19)	0.95 (0.55, 1.65)	1.82 (0.39, 10.44)		1.81 (0.32, 10.26)
≥6	92 (26.29)	258 (73.71)	0.84 (0.46, 1.53)	2.14 (0.32, 14.23)		2.33 (0.35, 15.49)
**Birth order**						
1	37 (29.84)	87 (70.16)	1	1		1
2-5	172 (27.61)	451 (72.39)	0.84 (0.49, 1.46)	0.51 (0.83, 3.12)		0.55 (0.09, 3.32)
≥6	111 (26.24)	312 (73.76)	0.77 (0.43, 1.38)	0.41 (0.58, 2.88)		0.42 (0.06, 2.93)
**Residence**						
Rural	271 (25.69)	784 (74.31)	1		1	1
Urban	49 (42.61)	66 (57.39)	3.69 (1.86, 7.31)		2.16 (1.05, 4.44)	0.96 (0.46, 1.99)
**Media exposure**						
Low	227 (24.70)	692 (75.30)	1		1	1
High	93 (37.05)	158 (62.95)	1.95 (1.31, 2.92)		1.52 (1.01, 2.29)	1.02 (0.66, 1.58)
**Region**						
Emerging	91 (18.61)	398 (81.39)	1		1	1
Developing	188 (30.62)	426 (69.38)	2.70 (1.68, 4.35)		2.75 (1.71, 4.46)	1.33 (0.80, 2.22)
City administration	41 (61.19)	26 (38.81)	18.10 (6.87, 47.6)		13.11 (4.96, 34.61)	5.69 (2.39, 13.61)^*^
**Distance to health facility**						
Big problem	176 (24.82)	533 (75.18)	1		1	1
Not big problem	144 (31.24)	317 (68.76)	1.42 (0.99, 2.04)		1.33 (0.92, 1.93)	0.99 (0.68, 1.44)

* Statistically significant at p-value < 0.05 in the full model (model 3).

AOR, Adjusted Odds Ratio; COR, Crude Odds Ratio; PNC, Postnatal care; Model 1: adjusted for individual-level factors, Model 2: adjusted for community-level factors, Model 3: adjusted for both individual and community-level factors (full model).

Hence, the odds of full immunization coverage among children 12–23 months of age from employed deviant mothers/caregivers was 1.69 higher than the odds of full immunization among children 12–23 months of age from non-employed deviant mothers/caregivers (AOR = 1.69, 95% CI: 1.68, 2.45). The odds of full immunization coverage among children aged 12–23 months of deviant mothers/caregivers from wealthy households were 2.54 higher than those from poor households (AOR = 2.54, 95%CI: 1.53, 4.22). The odds of full immunization coverage among children 12–23 months of age from deviant mothers/caregivers who reside in the city administration was 5.69 higher than that of deviant mothers/caregivers who reside in the emerging regions (AOR: 5.69, 95% CI: 2.39, 13.61). The odds of full immunization coverage among children 12–23 months of age from deviant mothers/caregivers who had 1–3 and greater or equal to four ANC follow up were 3.28 (AOR: 3.28, 95% CI: 2.12–5.07) and 3.91 (AOR: 3.91, 95% CI: 2.45, 6.24) higher than their counterparts, respectively.

## Discussion

This study identifies determinants that significantly increase the full immunization coverage among children 12–23 months of age from mothers/caregivers at high risk for low vaccine uptake (mothers/caregivers who do not have an education) in Ethiopia. Mothers/caregivers with no formal education are at particularly high risk for low immunization coverage among children aged 12–23 months and are consequently a key target group for improving childhood vaccine utilization. Identifying the positive determinants of PD behavior could be used to guide high-impact interventions to improve children's health in Ethiopia, where a significant proportion of mothers/caregivers are illiterate.

The overall full immunization coverage among children 12–23 months of age from deviant mothers/caregivers was 27.4% (95%CI: 25.0, 31.0). This finding is very low compared to previous studies estimates of full immunization coverage among children aged 12–23 months range from 36 to 77.4% in Ethiopia (1, 14, 17, 18, 23, 31), Kenya (40), Senegal (41), Burkina Faso (21), Nigeria (20), Indonesia (25), and Mozambique (22). This could be due to differences in outcome variable measurement, cultural practices, and study participants; for example, previous studies included both educated and uneducated mothers/caregivers, whereas our study only looked at mothers/caregivers with no formal education. Mothers/caregivers with no formal education may be unaware of the benefits of childhood immunization programs. Furthermore, mothers/caregivers without a formal education are less likely to engage in paid work and are more financially dependent on others. Even though immunization services have been provided freely, obtaining childhood immunization services still has an indirect cost. As a result, they may be unable to access these services for their children. On the other hand the differences in study periods, health system performance differences among countries, and the number of newly introduced vaccines like PCV and Rota incorporated into the definition of full expanded immunization program in Ethiopia can explain the variations (14).

The current study finding revealed that deviant mothers/caregiver's household wealth status, region, employment status, and ANC follow-up were statistically significant determinants of full immunization coverage among children 12–23 months of age from deviant mothers/caregivers and in concert with prior studies risk factor analysis (1, 14, 17, 18, 20, 31, 41).

We found that full immunization coverage among children 12–23 months of age from deviant mothers/caregivers who were employed was higher than those who do not employed. This finding agrees with the results of previous studies conducted in Ethiopia (14, 17), and Nigeria (20). This could be due to employed mothers/caregivers having better information about the benefits of childhood services including immunization, and also, they can cover the indirect cost of accessing the vaccine service. This implies that policymakers should create opportunities for mothers/caregivers to work and to become financially independent.

Our finding revealed that full immunization coverage among children 12–23 months of age from wealthy household deviant mothers/caregivers were higher than those with the poor wealth status supported with findings from other studies conducted Ethiopia (1, 6, 14, 17, 23), Indonesia (25), and Bangladesh (42). This could be inequalities in accessing healthcare between poor and wealthy households. Children from impoverished parents may face challenges in reaching health facilities compared to wealthy households (43); because low-income families had to incur high costs and take their time to maintain their daily lives. The other possible explanation could be that wealthier households have increased childcare practices and better health-seeking behavior (44). In contrast, studies conducted in Pakistan (45) revealed that household wealth status was not statistically associated with full immunization coverage. This could be because immunization is universal, and wealth no longer has as big of an impact on full immunization coverage as it formerly did because immunization offered through the EPI program is free, and public efforts to reach vulnerable mothers and children are continued (14). This implies that policymakers should continue to focus on developing interventions, initiatives, and expanding existing programs aimed at empowering women to develop their income and become economically self-sufficient. Consequently, their children will have better access to health services, including immunization.

In our study, full immunization coverage among children 12–23 months of age from deviant mothers/caregivers who reside in the city administration was higher than that of deviant caregivers who reside in the emerging regions. Similarly, studies conducted in Ethiopia showed that regions are significantly associated with full immunization coverage (6, 17). This could be due to regional differences in vaccine procurement, supply, cold chain, or other logistics issues (46, 47). This might also be due to differences in cultural beliefs, population size, topography, and levels of development, and this could be linked with differences in the availability of healthcare providers, vaccine and commodities. Hence, these regional differences tend to affect the range of childhood immunization uptake across the country (43). This implies that the government should improve infrastructure, i.e., electricity, transportation, and water, as well as health facilities, to increase the accessibility and uptake of full immunization among children in emerging regions.

In this study, fully immunization coverage among children 12–23 months of age from deviant mothers/caregivers who had 1–3 and greater or equal to four ANC follow up was higher than their counterparts. This finding is in line with the results of previous studies conducted in Ethiopia (1, 14, 17, 48, 49), Senegal (41), Nigeria (50), Uganda (51), Pakistan (45), and Indonesia (25). This could be because increased contact with a healthcare facility for ANC would promote full immunization coverage among children, giving mothers more opportunities to learn about child health, particularly the importance of vaccines, and be encouraged to immunized their children by healthcare professionals (52). This implies that policymakers should develop strategies to increase ANC service utilization by improving health facility accessibility and quality, and creating awareness through mass campaigns, which may ultimately increase the full immunization coverage.

### Strengths and limitations of the study

The positive deviance approach was used for the first time in Ethiopia to identify positive determinants for full immunization coverage among children 12–23 months of age from deviant mothers/caregivers. Non-educated mothers/caregivers are at high risk of their children's getting full immunization coverage so; identifying determinants using this positive deviance approach can improve the child health services policy in the country which can be transferable to other similar settings. In addition, multilevel analysis was employed to account for the hierarchical nature of the EDHS data to obtain reliable standard errors and estimates. Furthermore, because it is based on national survey data with large sample size, the study has the potential to give evidence for policymakers, program planners, and other stakeholders on how to create relevant interventions at both the national and regional levels to improve full immunization coverage among children. However, the findings of the study are interpreted with some limitations. We cannot show the temporal association between full immunization coverage among children aged 12–23 months of deviant mothers/caregivers and independent variables due to the cross-sectional nature of the survey. Since the EDHS survey relied on the respondents' reports, there might be a chance of recall bias.

## Conclusions

In Ethiopia, full immunization coverage among children 12–23 months of age from deviant mothers/caregivers is still low, and far behind the national target of full immunization coverage. The deviant mothers/caregiver's employment status, household wealth status, region, and ANC follow-up were associated with PD behavior and full immunization coverage. The positive deviance approach provides evidence for health policy makers and program implementers to identify determinants facilitating improved health behavior and, ultimately, better child health outcomes despite an acknowledged adverse risk profile. Such strategies and knowledge could facilitate targeted efforts aimed to improve child health outcomes and meet the national targets of child morbidity and mortality. Therefore, improving mothers/caregivers' employability, wealth status, and ANC follow-up will contribute to full immunization coverage improvement among children 12–23 months of age from deviant mothers/caregivers.

## Data availability statement

The datasets presented in this study can be found in online repositories. The names of the repository/repositories and accession number(s) can be found in the article/supplementary material.

## Ethics statement

Ethical review and approval was not required for the study on human participants in accordance with the local legislation and institutional requirements. Written informed consent for participation was not required for this study in accordance with the national legislation and the institutional requirements. Written informed consent was not obtained from the individual(s) for the publication of any potentially identifiable images or data included in this article.

## Author contributions

SM, DB, EF, and DA conceived the idea for this study. SM is involved in the data extraction, analysis, interpretation of the finding, and writing the original draft. DB and WD assisted in the analysis of the study. ES, RT, TA, HE, and FA writing the review and editing the manuscript. All the authors read and approved the final manuscript.
